# Ultrasound-Guided Greater Ischiatic Notch Plane Block Combined with the Caudal Quadratus Lumborum Block (GIN-TONIC Block) in Dogs Undergoing Pelvic Limb Surgery: Preliminary Results

**DOI:** 10.3390/ani14121764

**Published:** 2024-06-11

**Authors:** Pablo E. Otero, Jorge A. Guerrero, Lisa Tarragona, Fabiana Micieli, María Fernanda Sanchez, Pablo A. Donati, Martin R. Ceballos, Diego A. Portela

**Affiliations:** 1 Department of Anesthesiology and Pain Management, Facultad de Ciencias Veterinarias, Universidad de Buenos Aires, Buenos Aires C1427CWO, Argentina; jguer@fvet.uba.ar (J.A.G.); ltarragona@fvet.uba.ar (L.T.); mfsanchez@fvet.uba.ar (M.F.S.); pdonati@fvet.uba.ar (P.A.D.); mceballos@fvet.uba.ar (M.R.C.); 2 Department of Veterinary Medicine and Animal Production, University of Naples Federico II, 80137 Naples, Italy; fabiana.micieli@unina.it; 3 Department of Comparative, Diagnostic, and Population Medicine, College of Veterinary Medicine, University of Florida, Gainesville, FL 32610-0123, USA; dportela@ufl.edu

**Keywords:** analgesia, canine, nociception, regional anesthesia

## Abstract

**Simple Summary:**

The GIN-TONIC block is an interfascial ultrasound-guided regional anesthesia technique that desensitizes the lumbosacral plexus and provides analgesia to the pelvic limb. Different methods have been attempted previously, such as blocking specific plexuses (e.g., lumbar and sacral plexuses) and nerves (e.g., femoral and sciatic). However, these approaches either resulted in excessive motor block or failed to encompass all the required areas for surgery. This study assessed the efficacy of the GIN-TONIC block in dogs premedicated with either acepromazine or dexmedetomidine for surgeries on the hip and stifles. It also checked how well the dogs could move after the surgery. The results showed that the GIN-TONIC block provided adequate perioperative analgesia, independently of the drug used in premedication. During recovery, dogs showed minimal motor block, suggesting that GIN-TONIC block provides a motor-protective analgesic effect. However, more research is needed to fully understand how effective the GIN-TONIC block is during surgery.

**Abstract:**

This study assessed the analgesic and motor effects of the GIN-TONIC block, a combination of the greater ischiatic notch plane block and the caudal lateral quadratus lumborum block, in 24 dogs undergoing pelvic limb surgery. Dogs were randomly divided into two equal groups: G_A_ received acepromazine [(20 µg kg^−1^ intravenously (IV)] as premedication, and G_D_ received dexmedetomidine (2 µg kg^−1^ IV). General anesthesia was maintained with isoflurane, and both groups received a GIN-TONIC block using 2% lidocaine. Nociception during surgery and postoperative pain [assessed using the Glasgow Composite Measure Pain Score (GCMPS-SF)] were assessed. Fentanyl (2 µg kg^−1^ IV) was administered if nociception was noted and morphine (0.5 mg kg^−1^ IV) was administered during recovery if the pain scores exceeded the predefined threshold. Motor function was assessed during the recovery period using descriptors previously reported. All dogs received analgesics at the 4 h mark before being discharged. Three and two dogs in G_D_ and G_A_ required fentanyl once. Postoperative pain scores remained ≤4/20 for all dogs except one. Dogs achieved non-ataxic ambulation within 38.9 ± 10.3 and 35.1 ± 11.1 min after extubation in G_D_ and G_A_, respectively. This study highlighted the potential of the GIN-TONIC block as a feasible regional anesthesia method for delivering perioperative analgesia in dogs undergoing pelvic limb orthopedic surgery.

## 1. Introduction

Regional anesthesia has been integrated into the analgesic regimen for animals undergoing pelvic limb surgery [1]. Epidurals and perineural blocks have become standard practice among the frequently employed techniques [2,3,4,5,6,7]. However, a notable concern in applying these methods is the development of limb paralysis when concentrated local anesthetics are used [8], as well as the potential risk of neurological damage [9]. Furthermore, motor impairment in animals during recovery could result in instability and self-injury, and hinder early mobilization, a fundamental component of the enhanced recovery after surgery (ERAS) concept [10].

Interfascial plane blocks have emerged as a motor-sparing alternative for perineural blocks [11,12]. The preservation of motor function can be ascribed to the process of dilution and dispersion that the local anesthetic undergoes from its injection point into the interfascial space until it reaches the target nerves [13,14]. This mechanism results in uneven distribution along the target nerve, tending to favor the desensitization of small-diameter fibers (e.g., sensory A-delta and C-fibers) over large-diameter fibers (i.e., A-alpha motor fibers), which are responsible for nociception and motor conduction, respectively [15,16]. However, given that these blocks are usually integrated into a multimodal analgesic protocol that includes the systemic administration of analgesic drugs such as dexmedetomidine, methadone, ketamine, and nonsteroidal anti-inflammatory drugs (NSAID), among others, it remains crucial to evaluate the effectiveness of fascial plane blocks in mitigating the nociceptive response during surgical injury and facilitating a comfortable recovery without the supplementation of systemic analgesics.

A study involving healthy experimental dogs has introduced a novel combination of interfascial plane blocks aimed at desensitizing the lumbar and sacral plexuses [14]. This combination included the Greater Ischiatic Notch (GIN) plane block, designed to block the lumbosacral trunk [6], and the Caudal Quadratus Lumborum block (C-QLB), aimed at blocking the nerves of the lumbar plexus [14]. This combined technique, termed the GIN-TONIC block in this study, successfully desensitizes the tactile dermatomes of the thigh and stifle in non-premedicated dogs. Furthermore, compared with the perineural block of the lumbosacral plexus, the GIN-TONIC block resulted in a less pronounced motor block, suggesting an additional benefit in preserving a better motor function [14]. However, this approach has yet to be studied in animals undergoing pelvic limb surgery.

This pilot clinical trial aimed to assess the intraoperative hemodynamic response, postoperative pain scores, and motor function in client-owned dogs undergoing hip and stifle surgery receiving a GIN-TONIC block, utilizing 2% lidocaine in conjunction with dexmedetomidine or acepromazine administered intravenously as premedication and maintained under general anesthesia with isoflurane.

## 2. Materials and Methods

This prospective, pilot, randomized clinical trial was conducted following the Institutional Animal Care and Use Committee, Faculty of Veterinary Sciences, University of Buenos Aires (project no. 2023/18) approval and informed written consent obtained from each animal’s owner. Since the present study was designed as a pilot clinical trial, the number of animals per group was arbitrarily set at 12 based on similar studies [17,18]. Enrolled cases were, categorized as American Society of Anesthesiologists (ASA) ASA-II anesthetic risk, scheduled for elective hip or stifle surgeries from March to May 2022. The health status of the dogs was assessed via physical examinations and hematological and serum biochemistry evaluations. Inclusion criteria included dogs of any age, weight, size, or body condition score. Dogs displaying unrelated painful conditions, such as those related to osteoarthritis in other body regions, neurological deficits, or aggressive behavior were excluded. Before anesthesia, dogs underwent an 8 h fasting period for food and 2 h for water.

This study was designed to assess the intraoperative hemodynamic response, postoperative pain scores, and motor function in client-owned dogs undergoing unilateral pelvic limb surgery (i.e., femoral head excision, tibia-plateau-leveling-osteotomy, anterior cruciate ligament reconstruction with patellar tendon, or patellar luxation correction). All dogs received a combination of an ultrasound-guided GIN and C-QLB (i.e., GIN-TONIC) block using 2% lidocaine. Dogs were randomly assigned to receive intravenous (IV) premedication using either dexmedetomidine (Dexdomitor; Zoetis Ltd., Sao Paulo, Brazil) in Group G_D_ or acepromazine (Inadrin; Laboratorios Richmond, Buenos Aires, Argentina) in Group G_A_. Allocation concealment was ensured through opaque envelopes, and the assignment sequence was generated using a sequence generator (http://www.random.org/, accessed on 5 February 2024)

### 2.1. Anesthesia Protocol

Dogs were admitted two hours before general anesthesia and individually housed in separate kennels. A 20- or 22-gauge catheter was aseptically inserted into a cephalic vein to facilitate the subsequent administration of IV dexmedetomidine (2 µg kg^−1^; Group G_D_) or acepromazine (20 µg kg^−1^; Group G_A_). Following premedication, preoxygenation was conducted for five minutes using a fresh gas flow of 5 L min^−1^ via a facemask. Throughout the intraoperative period, IV administration of lactated Ringer’s solution was maintained at 3–5 mL kg^−1^ h^−1^. Antibiotic treatment with cephalotin (25 mg kg^−1^ IV; Cefalotina; Laboratorios Richet S.A., Buenos Aires, Argentina) was initiated at this stage in all animals. The induction of anesthesia was accomplished using IV propofol (Propovet, Richmond, Buenos Aires, Argentina) until achieving the absence of the palpebral reflex and a relaxed jaw tone. Subsequently, tracheal intubation was performed using an appropriately sized cuffed endotracheal tube and then connected to a sidestream capnograph and a circle breathing system coupled to an anesthetic machine (Advance CS2, General Electric, Springfield, IL, USA). The maintenance of anesthesia was accomplished through the vaporization of isoflurane in 100% oxygen. The isoflurane concentration was titrated to maintain a suitable plane of anesthesia within a range of end-tidal isoflurane concentrations (FE’Iso) of 1.1–1.3%, as determined by an anesthetist unaware of the assigned treatment group. Ventilation was controlled and adjusted to maintain end-tidal carbon dioxide (PE’CO_2_) between 35–45 mmHg. To prevent hypothermia, a heating blanket (Thermal Blanket Carbonvet cage, B. Braun Medical Inc., Shanghai, China) was used. Monitoring commenced immediately after premedication, encompassing continuous electrocardiogram monitoring of heart rate (HR) and rhythm, respiratory rate, PE’CO_2_, FE’Iso (%), peripheral hemoglobin oxygen saturation measured using a pulse oximetry probe on the tongue, core body temperature monitored via an esophageal probe, and oscillometric recordings of systolic, diastolic, and mean arterial pressure (MAP). Non-invasive blood pressure was measured at 3 min intervals with a suitably sized cuff placed around the right or left antebrachium just distal to the elbow. A pre-calibrated multi-parametric monitor (Digicare Lifewindow 9X, Digicare, Boynton Beach, FL, USA) was used to monitor. Five minutes after intubation, all animals received an ultrasound-guided GIN-TONIC block on the surgical limb. Anesthesia duration (defined as the total time of isoflurane administration), time from block execution to the start of surgery, surgery duration (measured from the first incision until completion of the last suture), extubation times, and sternal recumbency after isoflurane discontinuation were recorded for each dog.

### 2.2. Ultrasound-Guided Blocks

All approaches were performed following the aseptic preparation of the area by an experienced researcher using an ultrasound-guided technique (Sonosite M-Turbo and HLF-38; Sonosite Inc., Bothell, WA, USA). To perform the blocks, the dogs were placed in lateral recumbency with the limb to block uppermost.

**Figure 1:**
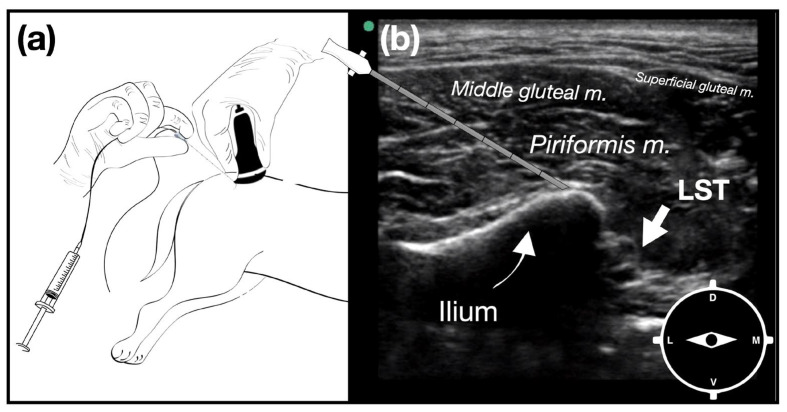
Ultrasound-guided greater ischiatic notch (GIN) plane injection. (**a**) The dog is positioned in lateral recumbency with the pelvic limbs in a neutral position. The transducer is positioned transversally on the middle third of an imaginary line connecting the cranial dorsal iliac spine with the medial angle of the ischiatic tuberosity, with its mark facing laterally. Ultrasound-guided GIN plane injection: the needle is introduced in-plane from the lateral aspect of the transducer. (**b**) Ultrasound image showing the dorsolateral aspect of the greater ischiatic notch and the neurovascular bundle containing the lumbosacral trunk (LST).

For the GIN plane block [6], the transducer was placed on the dorsal gluteal region, transversally to the ilium. When the dorsal aspect of the great ischiatic notch and the piriformis muscle were identified, a sonovisible needle (SonoPlex Stim cannula; Pajunk GmbH, Geisingen, Germany) was introduced in-plane in a latero-to-medial direction until it contacted the periosteum of the dorsomedial border of the greater ischiatic notch, where 0.2 mL kg^−1^ of 2% lidocaine (Lignocaina Gray 20 mg/mL; Productos Farmacéuticos Dr. Gray S.A.C.I., Buenos Aires, Argentina) was injected (Figure 1).

**Figure 2:**
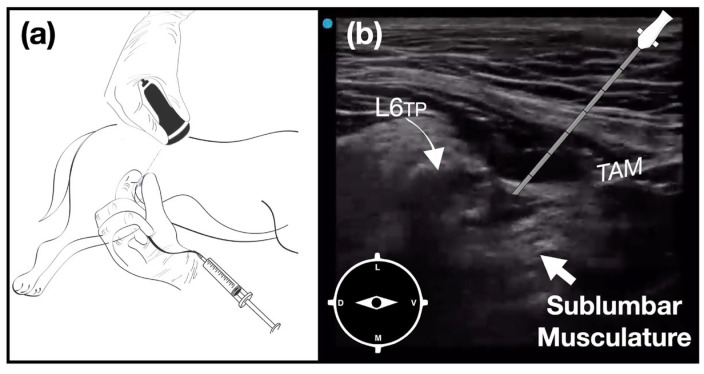
Ultrasound-guided caudal quadratus lumborum block (C-QLB). (**a**) The dog is positioned in lateral recumbency with the pelvic limbs in a neutral position. The transducer is positioned over the transverse process of L6 (L6TP), cranial and parallel to the crest of the ilium with its mark facing dorsal. (**b**) Ultrasound image showing the sublumbar musculature on the ventral aspect of the L6 transverse process. TAM: transversus adominis muscle.

For the C-QLB [14], the transducer was placed on the flank, cranial, and parallel to the crest of the ilium over the transverse process of the sixth lumbar vertebra (L6). Once the quadratus lumborum and psoas muscles were identified, the sonovisible needle was introduced in-plane in a ventral-to-dorsal direction until its tip reached the lateral aspect of the quadratus lumborum muscle, where 0.3 mL kg^−1^ of 2% lidocaine was injected (Figure 2).

The local anesthetic was injected after aspiration to reduce the risk of intravascular needle location in all cases. Interfascial distribution of the local anesthetic was confirmed during injection.

### 2.3. Intraoperative Period

All intraoperative evaluations were performed by a researcher blinded to the treatment group. Nociception was indirectly assessed through abrupt and clinically significant HR and MAP value shifts. Clinically significant changes were characterized as an increase of more than 25% in HR or MAP, sustained for over 2 min, compared with their respective values immediately prior to the abrupt rise [19]. In cases of nociception, a fentanyl (Fentanilo Gray; 50 µg/mL; Productos Farmacéuticos Dr. Gray S.A.C.I., Buenos Aires Argentina) bolus of 2 µg kg^−1^ IV was administered. If cardiovascular values did not revert to their baseline within 5 min, a continuous rate infusion (CRI) of fentanyl was initiated at 5 μg kg^−1^ h^−1^ and increased by 0.5 μg kg^−1^ h^−1^ every 5 min, until the variables returned to the pre-stimulation levels [18]. Changes in HR and MAP that did not meet analgesic rescue criteria were also recorded. If observed, the hemodynamic response was linked to specific actions: skin incision (TSI), joint capsule incision (TIC), joint distraction (TJD), bone drilling (TBD), if applicable, or skin suture (TSS), based on the corresponding time of recording. The block was deemed effective if the dog did not require any intraoperative analgesia or received up to 2 µg kg^−1^ of fentanyl, and ineffective if CRI of fentanyl was necessary [20].

In the event of hypotension, defined as a MAP < 65 mmHg, FE’Iso was reduced by 0.1%. If hypotension persisted despite an FE’Iso of 0.9%, an IV crystalloid bolus (10 mL kg^−1^ of normal saline over 15 min) was administered. If hypotension persisted, a second IV crystalloid bolus (5 mL kg^−1^ of normal saline over 5 min) was administered. Unresponsive hypotension was treated with a noradrenaline (Noradrenalina BIOL; Instituto Biológico Argentino S.A.I.C., Buenos Aires, Argentina) infusion starting at 0.1 µg kg^−1^ min^−1^ and increased by 0.1 mg kg^−1^ min^−1^ every 3 min until the MAP was above 65 mmHg. Bradycardia, defined as HR < 40 beats minute^−1^ for more than 1 min, was treated with atropine 0.02 mg kg^−1^ IV (Atropina HLB; HLB Pharma Group S.A., Buenos Aires, Argentina).

### 2.4. Postoperative Period

At the time of extubation, meloxicam (0.2 mg kg^−1^, subcutaneously; Meloxivet; John Martin SRL, Buenos Aires, Argentina) was administered to all dogs.

The Glasgow Composite Measure Pain Scale-Short Form (GCMPS-SF) [21] was employed for pain assessment. The evaluation was conducted by researchers proficient in using the scale and blinded to the treatment group, before anesthesia, once conscious sternal recumbency was achieved (T1) and 4 h after the execution of the GIN-TONIC block (T2). Rescue analgesia [0.5 mg kg^−1^ morphine, intramuscular (IM); Duramorph; Productos Farmaceuticos Dr Gray SACI, Buenos Aires, Argentina] was administered if the GCMPS-SF score reached ≥ 5/20.

The assessment of motor function in the blocked limb occurred once the animal resumed tripodal/quadrupedal non-ataxic ambulation, demonstrating weight-bearing and walking on a leash back and forth. The evaluation was conducted by an investigator unaware of group assignments.

The motor function was scored using a three-point scale for each descriptor: Normal (0): no evidence of block was detected. Reduced (1): evident compromise of the evaluated function. Absent (2): without action of the evaluated function. These observations included evaluating weight-bearing and gait pattern disturbance, which involved assessing paw proprioception and the motor function of muscles responsible for stifle joint flexion and extension, as well as adduction of the anesthetized limb and stability during walking.

This was study concluded after completing the final pain assessment (i.e., T2) or analgesic intervention. Subsequently, all the animals were transferred to the recovery area, where they received rescue analgesia and sedative drugs if needed until discharge. The dogs were discharged from the hospital on the day of surgery with a prescription of meloxicam (0.1 mg kg^−1^ QD orally) and gabapentin (12 mg kg^−1^ BID orally; Neurontin; Pfizer SRL, Buenos Aires, Argentina) for 5 days. All the animals were reevaluated the following day. If the pain scores had not reduced to below the rescue cut-off score, dipyrone (25 mg kg^−1^ BID orally; HLB Pharma Group S.A., Buenos Aires, Argentina) was added to the analgesic protocol. A follow-up period of one week was planned to evaluate any neurological deficits or side effects.

### 2.5. Statistical Analysis

As this is a preliminary pilot study devoid of hypotheses or sample size calculation, the gathered information is portrayed descriptively. Data analysis was conducted using GraphPad Prism Version 8.0 (GraphPad Software Inc., Solana Beach, CA, USA), with an evaluation for normality performed via the Shapiro–Wilk test. Normally distributed data are presented as mean ± standard deviation (SD), while non-normally distributed data are depicted as median (range). Categorical data are presented as proportions.

## 3. Results

Data from 24 ASA II client-owned dogs undergoing elective hip and stifle surgery were analyzed. Table 1 and Table 2 summarize type of surgery, demographic data, the time from block execution to the start of surgery, the duration of anesthesia and surgery, the time to extubation, and the times required to achieve sternal recumbency and non-ataxic ambulation in G_D_ and G_A_ groups. 

Induction of general anesthesia was achieved with 4.2 ± 1.4 mg kg^−1^ and 5.1 ± 1.6 mg kg^−1^ of propofol in the G_D_ and G_A_ groups, respectively. Median HR and MAP were 107 (77–124) beats minute^−1^ and 72 (62–109) mmHg for G_D_, and 102 (64–136) beats minute^−1^ and 64 (54–119) beats minute^−1^ for G_A_. The FE’Iso was 1.1% ± 0.2 in group G_D_ and 1.2% ± 0.1 in group G_A_. In group G_A_, one dog required a decrease in FE’Iso, and another needed an intravenous crystalloid bolus to address hypotension.

The block was judged effective in all the cases. Changes in HR or MAP not meeting the criteria for rescue analgesia at different time points are depicted in Table 3 and Table 4. Among G_D_ dogs, three out of twelve (25%) experienced nociceptive stimuli necessitating a single rescue dose of fentanyl. Reactions to surgical stimulation were noted in two during joint distraction and in one case in concomitance with bone drilling (Table 3). In group G_A_, two out of twelve (16.5%) animals required fentanyl once, one during joint distraction and the other while drilling the bone (Table 4). No hemodynamic changes were associated with skin incision or suturing in any of the cases.

None of the animals developed postanesthesia delirium/dysphoria. After the surgery, one animal in group G_A_ (case #7) received morphine (0.5 mg kg^−1^ IM) in response to a GCMPS-SF score of 8 at T1, and therefore it was subsequently excluded from the postoperative evaluation. In the remaining animals, the GCMPS-SF scores recorded at T1 (when conscious sternal recumbency was achieved, i.e., at 14.4 ± 4.5 and 11.6 ± 5.7 min in the G_D_ and G_A_ groups, respectively) and T2 (240 min after GIN-TONIC administration) remained ≤ 3/20 (Table 5). The 23 dogs that completed the study were transferred to the recovery area after the last GCMPS-SF score was recorded, which corresponded to 106.8 ± 28.7 and 110.0 ± 28.3 min from extubation in the G_D_ and G_A_ groups, respectively.

The motor function was scored at 136.3 ± 28.0 and 137.7 ± 23.7 min after the execution of the GIN-TONIC block and 38.9 ± 10.3 and 35.1 ± 11.1 min after extubation in the G_D_ and G_A_ groups, respectively (Table 6 and Table 7). After surgery, four dogs in the G_D_ group and three dogs in the G_A_ group showed partial or complete loss of function of the quadriceps muscle. Both groups had four dogs with partial or complete loss of proprioception in the blocked limb. Furthermore, one dog from each group had marked hyperadduction (i.e., no evidence of block of the obturator nerve) of the blocked limb.

No neurological complications or cutaneous alterations at the injection site were observed during the 7-day follow-up period.

## 4. Discussion

The current preliminary pilot study demonstrated the effectiveness of the interfascial plane GIN-TONIC block using 2% lidocaine for providing intraoperative antinociception and early postoperative analgesia in dogs undergoing hip and stifle surgery, regardless of the intravenous premedication with dexmedetomidine or acepromazine.

Dexmedetomidine is a systemic sedative and analgesic in dogs [22]. Multiple studies have confirmed that the systemic administration of dexmedetomidine, either through a single intramuscular injection or constant rate intravenous infusion, significantly prolongs the duration of regional anesthesia and improves postoperative pain relief following perineural lidocaine administration for stifle surgery in dogs [23,24]. However, when comparing the efficacy of perineural femoral and sciatic nerve blocks using 2% lidocaine in dogs undergoing stifle surgery, premedicated with acepromazine, to those receiving systemic dexmedetomidine, no discernible differences were observed between the groups regarding the incidence of nociceptive events requiring analgesic intervention [23,24]. These findings confirm the role of nerve block in the overall analgesic effect in dogs undergoing stifle surgery. Our results align with these reports, similarly showing the role of the GIN-TONIC block in pelvic limb surgeries, including hip and stifle joints. Consistent findings regarding the effectiveness of perineural blocks were documented in dogs receiving long-acting local anesthetics such as bupivacaine and ropivacaine, premedicated with dexmedetomidine alongside opioids such as methadone, morphine, or hydromorphone [19,20,25,26,27,28]. Nonetheless, despite their efficacy, a variable proportion of animals required at least one intraoperative analgesic rescue. Remarkably, the incidence of animals necessitating intraoperative analgesic intervention during pelvic limb surgery remained consistent, irrespective of the premedication regimen or perineural block approach utilized.

Our findings suggest that the occurrence of cardiovascular response to nociceptive events was comparable between animals premedicated with acepromazine or dexmedetomidine, aligning with previously published data [19,20,24]. This reinforces the role of the GIN-TONIC block as a potential regional anesthetic technique for dogs undergoing pelvic limb surgeries.

In this study, the block was deemed effective if the dog did not require intraoperative analgesia or received up to 2 µg kg^−1^ of fentanyl. However, less than 25% self-limiting increases in heart rate HR or MAP were observed in response to stimuli that did not meet the criteria for rescue analgesia. These responses, considered tolerable because they do not significantly alter the reference hemodynamic parameters (i.e., HR and MAP), could be related to an incomplete or shallow block. It has been observed that the bioavailability of local anesthetic in the axoplasm of the affected nerves is lower for interfascial blocks as compared with perineural blocks, which could explain the fluctuations seen while applying high-intensity stimuli like those involved in joint distraction and bone drilling [13,14,16]. However, the scarce magnitude of the changes and the absence of signs of pain or discomfort in the animals during recovery confirm the established cut-off point for intraoperative analgesic rescue as acceptable. Additionally, pain scores recorded during recovery provide evidence of adequate sensory blockade, at least up to 4 h after the block was performed. However, further investigations are necessary to clarify this matter.

The pelvic limb’s motor input originates from the lumbar and sacral plexuses [29]. The fact that perineural blocks of the lumbosacral plexus promote a marked motor deficit has prompted the advancement of motor-sparing techniques, such as the combination of the saphenous and sciatic nerve blocks for stifle surgery [30]. However, perineural blocks performed distal to the lumbosacral plexus are characterized by the lack of blocks on articular branches derived from motor nerves such as the femoral and obturator [31,32]. Furthermore, distal blocks do not block the cutaneous branches of the nerves responsible for dermatomes of the thigh [8]. Interestingly, the GIN-TONIC block provides a broader coverage of limb tissues, including dermatomes, as noted by the lack of response in coincidence with skin incision and suture.

In our study, most dogs displayed a low-intensity motor deficit, enabling early mobilization in the immediate postoperative period while maintaining a stable tripodal gait. This is partly attributed to the less intense motor blockade facilitated by the interfacial blocks and the preservation of muscle function responsible for body stability [13,14,16]. However, these results may be influenced by using lidocaine, a short-acting anesthetic [23,24]. Future studies using long-acting local anesthetics such as bupivacaine or ropivacaine are necessary.

Despite showing motor sparing effect in most cases, some animals showed signs of femoral and sciatic nerve block. Furthermore, in certain animals, the obturator block proved ineffective. The absence of an obturator nerve block, combined with the lumbosacral trunk block preventing abduction forces of the biceps femoris and gluteus medius muscles, led to significant hyperadduction of the limb, compromising gait stability during ambulation, even in a tripodal stance [19]. In this context, the C-QLB emerges as a promising alternative to a psoas compartment approach, providing a limited motor block and efficient obturator nerve block within the retropsoas compartment [14]. However, further investigations employing long-acting anesthetics such as bupivacaine or ropivacaine are necessary to elucidate this innovative technique’s clinical utility.

According to Murali and Charlesworth [33], pilot studies serve various purposes, including evaluating study protocols and determining sample size. Moreover, experts advise that pilot study analyses should predominantly be descriptive, as hypothesis testing typically requires a larger sample size and a control arm, elements often absent in these smaller-scale investigations [34]. Nevertheless, the results obtained in our study offer valuable insights. Firstly, the GIN-TONIC block effectively mitigated nociception and facilitated early pain relief throughout the local anesthetic’s action period in all but one of the tested dogs. Secondly, these findings contribute further evidence to the role of the GIN-TONIC block in managing pain in dogs undergoing pelvic limb surgery. Thirdly, the observed pain relief during and after surgery sheds light on the applicability of this approach to pelvic limb surgeries. Comparable outcomes were reported with the combination of the GIN plane block and the lateral preiliac approach in pelvic limb surgeries for dogs [28]. Nonetheless, given the limited sample size in our study, a comprehensive investigation is warranted. Nevertheless, when a motor-protective analgesic strategy is desired, the GIN-TONIC block presents an attractive option for administering a lumbosacral plexus block.

This study presents several limitations that warrant discussion and consideration when interpreting the findings. Firstly, the small sample size, determined by the pilot nature of the investigation and the allocation of only 12 animals per group, may restrict the generalizability of the results and limit the statistical power to detect nuanced differences or associations. Additionally, the exclusion criteria, which omitted dogs with specific conditions such as unrelated painful conditions, neurological deficits, or hostile behavior, may introduce selection bias and constrain the extrapolation of findings to a broader population of animals. Moreover, this study’s focus on a specific set of elective hip and stifle surgeries may restrict the applicability of the findings to other surgical procedures.

The limited follow-up period of 4 h postoperatively may overlook delayed complications or the long-term effects of the anesthesia and analgesic techniques employed. However, due to ethical considerations and the imperative to ensure animal welfare, we implemented an analgesic rescue protocol timed to coincide with the expected duration of the local anesthetic used [23,24]. This decision was made to provide timely pain relief while minimizing the risk of prolonged discomfort or distress for the animals involved in this study.

Operator dependency in performing ultrasound-guided blocks may lead to variability in block efficacy and reproducibility when applied by less experienced operators. The controlled experimental setting and the use of client-owned dogs undergoing elective surgeries at a specific institution may limit the external validity of the study’s findings, hindering their application to real-world clinical scenarios. Additionally, the evaluation of outcome measures was primarily focused on intraoperative hemodynamic response, postoperative pain scores, and motor function, lacking additional objective measures such as biochemical markers of stress (e.g., cortisol, catecholamines, lactate, glucose, and IL) or muscle function assessments, which could provide a more comprehensive understanding of the interventions’ effects [35]. Therefore, these limitations should be considered when interpreting this study’s results and designing future research endeavors.

## 5. Conclusions

In summary, this pilot study highlighted the potential of the GIN-TONIC block as a feasible regional anesthesia method for delivering perioperative analgesia in dogs undergoing pelvic limb orthopedic surgery. However, further clinical exploration, including prospective randomized controlled trials, is necessary to solidify the efficacy of the GIN-TONIC plane block in this context.

## Figures and Tables

**Table 1:** Demographic data, time from block execution to the start of surgery (TB-S), duration of anesthesia and surgery, time to extubation, and times required to achieve sternal recumbency and non-ataxic ambulation in dogs undergoing hip and stifle surgery, treated with a GIN-TONIC block, using 2% lidocaine, premedicated with dexmedetomidine and maintained under general anesthesia with isoflurane (G_D_ group). The results are presented as mean and standard deviation (SD) for all variables except for body score condition (BSC), which is reported as median (range).

Case (Surgery)	Breed	Weight (kg)	Age (Years)	BCS	Sex	TB-S (min)	Duration of Anesthesia (min)	Duration of Surgery (min)	Time to Extubation (min)	Time to Achieve Sternal Recumbency (min)	Time to Achieve Non-Ataxic Ambulation (min)
1 (FHE)	Poodle	2.5	6	5	♂	17	76	36	12	15	50
2 (FHE)	Maltese	5	0.5	5	♀	15	70	35	6	12	32
3 (TPLO)	Boxer	29	3	6	♂	20	136	98	5	19	38
4 (FHE)	Mongrel	18	5	6	♀	15	105	60	7	13	35
5 (FHE)	Poodle	6	9	8	♀	21	75	30	11	13	28
6 (ACLR)	Mongrel	18	6	6	♀	15	113	83	4	7	31
7 (ACLR)	Poodle	2.9	4	5	♀	15	108	60	20	25	40
8 (TPLO)	Pitbull	29.7	7	6	♀	15	120	73	7	15	45
9 (TPLO)	Pitbull	33	3	7	♂	12	165	135	9	10	30
10 (PLC)	F-Bulldog	6.6	2	7	♂	25	90	30	12	15	50
11 (PLC)	Poodle	3	3	7	♀	15	70	35	10	15	60
12 (TPLO)	Labrador	37	4	8	♀	18	100	55	10	14	28
MEAN (SD)	-	15.9 (13.2)	4.4 (20.3)	-	-	16.9 (3.6)	102.3 (28.9)	60.8 (32.2)	9.4 (4.3)	14.4 (4.5)	38.9 (10.3)
MEDIAN (range)	-	-	-	6 (5.8–7.0)	-	-	-	-	-	-	-

FHE: femoral head excision; TPLO: tibia-plateau-leveling-osteotomy; ACLR: anterior cruciate ligament reconstruction with patellar tendon; PLC: patellar luxation correction.

**Table 2:** Demographic data, time from block execution to the start of surgery (TB-S), duration of anesthesia and surgery, time to extubation, and times required to achieve sternal recumbency and non-ataxic ambulation in dogs undergoing hip and stifle surgery, treated with a GIN-TONIC block, using 2% lidocaine, premedicated with acepromazine and maintained under general anesthesia with isoflurane (G_A_ group). The results are presented as mean and standard deviation (SD) for all variables except for body score condition (BSC), which is reported as median (range).

Case (Surgery)	Breed	Weight (kg)	Age (Years)	BCS	Sex	TB-S (min)	Duration of Anesthesia (min)	Duration of Surgery (min)	Time to Extubation (min)	Time to Achieve Sternal Recumbency (min)	Time to Achieve Non-Ataxic Ambulation (min)
1 (TPLO)	Pitbull	27	3	7/9	♀	15	105	75	6	7	30
2 (FHE)	Poodle	7.2	7	5/9	♀	14	62	27	10	24	45
3 (TPLO)	Mongrel	32	8	8/9	♀	20	134	90	5	6	20
4 (PLC)	Mongrel	4.8	5	6/9	♂	15	98	60	5	5	28
5 (ACLR)	Beagle	11.9	3	7/9	♂	15	90	50	7	10	60
6 (TPLO)	Mongrel	23.5	6	5/9	♂	19	155	110	3	12	25
7 (ACLR)	Collie	21	3	5/9	♂	14	85	55	6	18	35
8 (ACLR)	Poodle	5	2	8/9	♂	15	110	75	10	12	26
9 (FHE)	Beagle	9.6	5	7/9	♀	20	140	85	8	10	40
10 (PLC)	Poodle	5.2	1	5/9	♂	14	76	45	4	5	45
11 (ACLR)	Pug	13	7	8/9	♀	25	125	70	15	15	30
12 (FHE)	Mongrel	33	4	7/9	♀	18	90	45	10	15	37
MEAN (SD)	-	16.1 (10.7)	4.5 (2.2)		-	17.0 (3.4)	105.8 (27.9)	65.6 (23.1)	7.4 (3.4)	11.6 (5.7)	35.1 (11.1)
MEDIAN (range)	-	-	-	7 (5.0–7.2)	-	-	-	-	-	-	-

FHE: femoral head excision; TPLO: tibia-plateau-leveling-osteotomy; ACLR: anterior cruciate ligament reconstruction with patellar tendon; PLC: patellar luxation correction.

**Table 3:** Changes in heart rate (HR) and mean arterial blood pressure (MAP) and its relationship with the surgical stimuli [i.e., skin incision (TSI), joint capsule incision (TIC), joint distraction (TJD), bone drilling (TBD), skin suture (TSS)], in dogs undergoing hip and stifle surgery, treated with a GIN-TONIC block, using 2% lidocaine, premedicated with dexmedetomidine and maintained under general anesthesia with isoflurane (G_D_ group). Nociception was defined as an increase >25% in HR or MAP, sustained for over 2 min, compared with their respective values immediately prior to the abrupt rise.

Case (Surgery)	Increases in HR from 1 to 15%	Increases in PAM from 1 to 15%	Increases in HR from 11 to 25%	Increases in PAM from 11 to 25%	Increases in HR >25%	Increases in PAM >25%
1 (TPLO)	0	0	0	0	0	0
2 (FHE)	1 (TIC)	1 (TIC)	0	0	0	0
3 (TPLO)	1 (TJD)	1 (TJD)	0	1 (TBD)	1 (TBD)	1 (TBD)
4 (PLC)	0	0	0	0	0	0
5 (ACLR)	0	0	0	0	0	0
6 (TPLO)	1 (TIC)	1 (TIC)	1 (TJD)	1 (TJD)	1 (TJD)	1 (TJD)
7 (ACLR)	0	0	0	0	0	0
8 (ACLR)	1 (TSI)	0	0	0	0	1 (TJD)
9 (FHE)	0	0	0	0	0	0
10 (PLC)	0	0	0	0	0	0
11 (ACLR)	0	0	0	1 (TIC)	0	0
12 (FHE)	0	0	1 (TJD)	1 (TJD)	0	0

FHE: femoral head excision; TPLO: tibia-plateau-leveling-osteotomy; ACLR: anterior cruciate ligament reconstruction with patellar tendon; PLC: patellar luxation correction. 0: no response was detected during surgical injury; 1: response was detected during (type of surgical stimuli).

**Table 4:** Changes in heart rate (HR) and mean arterial blood pressure (MAP) and its relationship with the surgical stimuli [i.e., skin incision (TSI), joint capsule incision (TIC), joint distraction (TJD), bone drilling (TBD), skin suture (TSS)], in dogs undergoing hip and stifle surgery, treated with a GIN-TONIC block, using 2% lidocaine, premedicated with acepromazine and maintained under general anesthesia with isoflurane (G_A_ group). Nociception was defined as an increase >25% in HR or MAP, sustained for over 2 min, compared with their respective values immediately prior to the abrupt rise.

Case (Surgery)	Increases in HR from 1 to 15%	Increases in PAM from 1 to 15%	Increases in HR from 11 to 25%	Increases in PAM from 11 to 25%	Increases in HR >25%	Increases in PAM >25%
1 (TPLO)	1 (TJD)	1 (TJD)	0	0	0	0
2 (FHE)	0	0	0	0	0	0
3 (TPLO)	0	0	0	0	0	0
4 (PLC)	0	0	1 (TIC)	1 (TIC)	0	0
5 (ACLR)	1	1	1	1	0	0
6 (TPLO)	1	0	0	0	0	0
7 (ACLR)	1 (TIC)	1 (TIC)	0	1 (TBD)	0	1 (TBD)
8 (ACLR)	0	0	0	0	0	0
9 (FHE)	0	0	1 (TJD)	0	1 (TJD)	1 (TJD)
10 (PLC)	1	1	0	0	0	0
11 (ACLR)	1 (TJD)	1 (TJD)	0	1 (TBD)	0	0
12 (FHE)	0	0	0	0	0	0

FHE: femoral head excision; TPLO: tibia-plateau-leveling-osteotomy; ACLR: anterior cruciate ligament reconstruction with patellar tendon; PLC: patellar luxation correction. 0: no response was detected during surgical injury; 1: response was detected during (type of surgical stimuli).

**Table 5:** Pain scores as median (range) for the 24 animals randomly assigned to receive dexmedetomidine (G_D_) or acepromazine (G_A_) as premedication as part of the anesthetic protocol in dogs scheduled for hip and stifle surgeries. These animals were treated with a GIN-TONIC block using 2% lidocaine and maintained under general anesthesia with isoflurane. Pain scores were assessed before anesthesia (T0), once conscious sternal recumbency was achieved (T1) and 4 h after the execution of the GIN-TONIC block (T2).

Case	GCMPS-SF G_D_			Case	GCMPS-SF G_A_		
	T0	T1	T2		T0	T1	T2
1 (FHE)	0	0	0	1 (TPLO)	0	0	0
2 (FHE)	1	3	2	2 (FHE)	2	0	1
3 (TPLO)	0	0	0	3 (TPLO)	0	0	0
4 (FHE)	0	0	0	4 (PLC)	0	1	3
5 (FHE)	0	0	0	5 (ACLR)	0	0	0
6 (ACLR)	0	1	1	6 (TPLO)	0	0	0
7 (ACLR)	0	0	0	7 (ACLR)	0	8	–
8 (TPLO)	0	2	1	8 (ACLR)	0	2	2
9 (TPLO)	2	0	0	9 (FHE)	1	0	0
10 (PLC)	0	0	0	10 (PLC)	0	2	1
11 (PLC)	0	1	1	11 (ACLR)	0	0	0
12 (TPLO)	0	0	0	12 (FHE)	0	0	0
MEDIAN (range)	0 (0–2)	0 (0–3)	0 (0–2)	MEDIAN (range)	0 (0–2)	0 (0–8)	0 (0–3)

GCMPS-SF, Glasgow Composite Measure Pain Scale. FHE: femoral head excision; TPLO: tibia-plateau-leveling-osteotomy; ACLR: anterior cruciate ligament reconstruction with patellar tendon; PLC: patellar luxation correction.

**Table 6:** Assessment of motor function in 12 dogs undergoing hip and stifle surgery, premedicated with dexmedetomidine (G_D_ group), upon achieving non-ataxic ambulation, 136.3 ± 28.0 min after the execution of the GIN-TONIC block with 2% lidocaine. Normal (0): no evidence of block was detected. Reduced (1): evident compromise of the evaluated function. Absent (2): without action of the evaluated function.

Case (Surgery)	Stifle Joint Extension of the Blocked Limb	Stifle Joint Flexion of the Blocked Limb	Adduction of the Blocked Limb	Paw Proprioception of the Blocked Limb	Weight-Bearing of the Blocked Limb	Stability During Walking
1 (FHE)	0	0	2	0	1	0
2 (FHE)	0	0	2	0	0	0
3 (TPLO)	2	0	2	1	2	0
4 (FHE)	0	0	2	0	1	0
5 (FHE)	2	1	2	1	2	2
6 (ACLR)	1	1	2	1	1	0
7 (ACLR)	2	2	2	2	2	2
8 (TPLO)	0	1	0	0	1	0
9 (TPLO)	0	0	2	0	1	0
10 (PLC)	0	0	2	0	1	0
11 (PLC)	0	0	2	0	0	0
12 (TPLO)	0	0	2	0	1	0
MEDIAN (range)	0 (0–2)	0 (0–2)	2 (0–2)	0 (0–2)	1 (0–2)	0 (0–2)

FHE: femoral head excision; TPLO: tibia-plateau-leveling-osteotomy; ACLR: anterior cruciate ligament reconstruction with patellar tendon; PLC: patellar luxation correction.

**Table 7:** Assessment of motor function in 12 dogs undergoing hip and stifle surgery, premedicated with acepromazine (G_A_ group), upon achieving non-ataxic ambulation, 137.7 ± 23.7 min after the execution of the GIN-TONIC block with 2% lidocaine. Normal (0): no evidence of block was detected. Reduced (1): evident compromise of the evaluated function. Absent (2): without action of the evaluated function.

Case (Surgery)	Stifle Joint Extension of the Blocked Limb	Stifle Joint Flexion of the Blocked Limb	Adduction of the Blocked Limb	Paw Proprioception of the Blocked Limb	Weight-Bearing of the Blocked Limb	Stability when Walking
1 (TPLO)	0	0	2	0	1	0
2 (FHE)	0	0	2	0	1	0
3 (TPLO)	2	1	0	1	2	2
4 (PLC)	0	0	2	0	1	0
5 (ACLR)	0	0	2	0	1	0
6 (TPLO)	2	0	2	0	2	0
7 (ACLR)	–	–	–	–	–	–
8 (ACLR)	1	2	2	2	1	0
9 (FHE)	0	0	2	0	1	0
10 (PLC)	0	0	1	0	2	0
11 (ACLR)	0	1	2	1	1	0
12 (FHE)	0	1	2	1	1	0
MEDIAN (range)	0 (0–2)	0 (0–2)	2 (0–2)	0 (0–2)	1 (1–2)	0 (0–2)

FHE: femoral head excision; TPLO: tibia-plateau-leveling-osteotomy; ACLR: anterior cruciate ligament reconstruction with patellar tendon; PLC: patellar luxation correction.

## Data Availability

The data presented in this study are available on request from the corresponding author. The data are not publicly available due to confidential identification of the animals.
